# Laparoscopic radical surgery for locally advanced T4 transverse colon cancer and prognostic factors analysis: Evidence from multi-center databases

**DOI:** 10.1097/MD.0000000000036242

**Published:** 2023-12-01

**Authors:** Feng Xie, Pingfan Lu, Yuming Chen, Xiangjun Liu, Zhenhong Zou, Jinheng Gan

**Affiliations:** a Department of General Surgery, The Second Affiliated Hospital of Nanchang University, Jiangxi, People’s Republic of China.

**Keywords:** laparoscopic, pT4, radical resection, transverse colon cancer

## Abstract

The safety and efficacies of laparoscopic radical procedures are still controversial for locally advanced pathological T4 (pT4) TCC (transverse colon cancer). Therefore, the aim of this study is to evaluate the oncologic and perioperative outcomes and to recognize the prognostic factors in radical resection for pT4 TCC derived from multi-center databases. 314 patients with TCC who underwent radical resection between January 2004 and May 2017, including 139 laparoscopic resections and 175 open resections, were extracted from multicenter databases. Oncological as well as perioperative outcomes were investigated. The baseline characteristics of the 2 groups did not differ significantly. Nevertheless, the laparoscopic technique was found to be linked with a significantly longer duration of surgery (208.96 vs 172.89 minutes, *P* = .044) and a significantly shorter postoperative hospital stay (12.23 vs 14.48 days, *P* = .014) when compared to the conventional open approach. In terms of oncological outcomes, lymph node resection (16.10 vs 13.66, *P* = .886), 5-year overall survival (84.7% vs 82.7%, *P* = .393), and disease-free survival (82.7% vs 83.9%, *P* = .803) were similar between the 2 approaches. Based on multivariate analysis, it was determined that differentiation and N classification were both independent prognostic factors for overall survival. However, it was found that only N classification was an independent prognostic factor for disease-free survival. These findings underscore the significance of differentiation and N classification as key determinants of patient outcomes in this context. Overall, the laparoscopic approach may offer advantages in terms of shorter hospital stays, while maintaining comparable oncological outcomes. Laparoscopic radical procedure can gain a couple of short-term benefits without reducing long-term oncological survival for patients with pT4 TCC.

## 1. Introduction

Colorectal cancer is one of the leading causes of death from all malignant tumors with an estimated 135,430 new cases and 50,260 deaths during 2017.^[1]^ Laparoscopic colectomy has been widely accepted for resection of colorectal cancer, with a lower incidence of short-term complication and similar long-term outcomes in stage I-III patients,^[2–5]^ compared to open colectomy. Despite advances in cancer treatment, a subset of patients diagnosed with locally advanced cancer (T4) with locoregional invasion may have a poor prognosis, with approximately 10% of patients falling into this category.^[6]^ For locally advanced colorectal cancer, this tumor has a reasonable chance of cure when accompanied by a combined multi-organ resection,^[7–10]^ but in a landmark randomized study locally advanced colon cancer was excluded when comparing laparoscopic colectomy with open colectomy.^[11]^ The safety and efficacy of laparoscopic colectomy in patients with T4 colon cancer remains a topic of debate in the medical community. One of the primary reasons for this is the technical difficulties associated with en-bloc resection of large tumors, which can make it challenging to achieve optimal outcomes. On the one hand, The American Society of Colon & Rectal Surgeons has issued recommendations regarding the use of laparoscopic resection in cases of T4 colon cancer. According to their guidelines, this approach can be performed safely and effectively, with no significant difference in long-term oncologic outcomes when compared to open surgery.^[12]^ On the other hand, the National Comprehensive Cancer Network guidelines for colon cancer surgery recommend laparoscopic colectomy should not be used in locally advanced disease.^[13]^ The European Association of Endoscopic Surgery (EAES) also did not to recommend laparoscopic colectomy in locally advanced colon cancer.^[14]^ Recent several researches have compared laparoscopic colectomy with open colectomy in locally advanced colon cancer,^[15–18]^ but many of those researches studied patients from single institutions or only used small sample sizes.

Transverse colon malignant tumors were also precluded from these previous trials owing to several reasons. First of all, the operative technique and extent of standard lymph node dissection may vary depending on the specific location of the malignant tumor. This underscores the importance of tailoring surgical approaches to account for the unique anatomical characteristics and clinical features of each individual case. Furthermore, the laparoscopic resection of TCC demands a high degree of surgical precision, particularly given the technical complexity involved in performing lymph node dissection around the middle colic vessels. Furthermore, the presence of anatomical variations associated with these vessels further underscores the need for meticulous surgical skill and expertise in this context. Finally, because of malignancies of the transverse colon account for only 10% of all colon malignancies, surgeons have limited experience in treating them.

With the advancement of laparoscopic equipment and the growing expertise in laparoscopic surgery, numerous studies have been conducted to evaluate the safety and effectiveness of the laparoscopic approach for the resection of T4 malignancies in the transverse colon.^[19]^ Therefore, the aim of this investigation was to evaluate the short- and long-term outcomes of laparoscopic radical surgery for pT4 TCC, with a particular emphasis on identifying the prognostic factors that are associated with this surgical approach. Through a thorough analysis of the clinical data and outcomes of patients who underwent laparoscopic resection for pT4 TCC, we aimed to enhance our comprehension of the safety and efficacy of this technique in the management of advanced colon cancer.

## 2. Materials and methods

The objective of this retrospective investigation was to compare the clinical outcomes of TCC patients who underwent laparoscopic or open potential radical resection and were diagnosed with pT4a-4bN0-3M0 according to the 7th edition of the American Joint Committee on Cancer Staging System. Our objective was to comprehensively evaluate the advantages and disadvantages of laparoscopic surgery versus open surgery in the management of advanced colon cancer by scrutinizing the data derived from these patients. The study was conducted utilizing the TCC database at 3 Nanchang University-affiliated hospitals, spanning the time frame from January 2004 to May 2017. In this study, we scrutinized patient demographics, post-operative complications, overall survival (OS), and disease-free survival (DFS). The research procedures were in strict accordance with the principles outlined in the Declaration of Helsinki. Additionally, approval for the study was granted by the Ethics Committee at the Second Affiliated Hospital of Nanchang University, ensuring that all aspects of the study were conducted in a manner that was both ethical and scientifically rigorous.

### 2.1. Surgical indications and procedures

In preoperative diagnosis and clinical stage, endoscopic biopsy is often combined with abdominal computed tomography. In instances where tumors were detected at the liver flexure, the preferred surgical intervention was right hemicolectomy, whereas tumors located at the splenic flexure were typically managed with left hemicolectomy. By tailoring the surgical approach to the specific location of the tumor, we aimed to optimize patient outcomes and minimize the risk of complications. For tumors located between these 2 sites, a transverse colectomy was performed. Right hemicolectomy involved ligation at the origins of the right branch of the middle colic vessels, right colic vessels, and ileocolic vessels, along with lymph node dissection. In the course of performing an expanded right hemicolectomy, the surgeon first ligated the origins of the middle colic, ileocolic, and right colic vessels. Subsequently, the ileum, cecum, ascending colon, and hepatic flexure were carefully resected in a manner that was designed to minimize the risk of complications and optimize patient outcomes. Left hemicolectomy is a surgical procedure that involves the careful ligation of the left branch of the middle colic vessels and the left colic vessels, as well as lymph node dissection and resection of the descending colon and sigmoid colon. The surgical procedure of extended left hemicolectomy includes ligation of both the middle colic vessels and the origin of left colonic vessels, followed by resection of approximately one-third of the transverse colon, the descending colon, and a portion of the sigmoid colon. In addition, the procedure includes meticulous lymph node dissection. The surgical resection of the transverse colon requires meticulous ligation at the origins of the middle colic vessels, coupled with a comprehensive lymph node dissection. The selection of the surgical approach was made based on the discretion of the operating surgeon. The conversion from laparoscopic to open surgery was defined as an unintended abdominal incision that was larger than the incision required for the planned specimen extraction. The patients were closely monitored and subjected to a follow-up schedule of every 3 months during the initial 2 years post-surgery, followed by every 6 months until the fifth year. Follow-up visits were conducted annually after the 5-year mark, with the most recent follow-up completed in July 2018. The follow-up data were collected from various sources, including medical records, telephone communication, and home visits.

### 2.2. Statistical analysis

Continuous variables that conformed to a normal distribution were reported as mean ± standard deviation, and categorical variables were presented as median (range). The evaluation of OS and DFS rates was conducted using the Kaplan–Meier method, while the Log-rank test was employed to compare prognostic factors and survival rates. The Cox proportional hazards model was utilized for the univariate and multivariate analyses. The results were reported as risk hazard ratio (HR) with a 95% confidence interval (95% CI). Statistical significance was set at *P* < .05 (two-tailed). The data analysis was performed using SPSS (version 21.0; SPSS Inc., Chicago, IL, USA) to ensure the statistical analysis was rigorous and reliable. These methods were implemented to ensure the robustness and reliability of the study findings.

## 3. Results

The study included a total of 314 patients with pT4 TCC who were considered eligible for potential radical resection. Of these, 175 (55.7%) underwent open operation, while 139 (44.3%) underwent laparoscopic operation. The demographic characteristics of both groups were similar, as demonstrated in Table 1. Specifically, there were no statistically significant differences observed in terms of age, gender, weight, ASA score, history of previous abdominal surgery, and type of previous surgery. These results suggest that the 2 groups were comparable in terms of patient characteristics, which is important for minimizing any potential confounding factors in the analysis.

**Table 1:** Characteristics of patients (n = 314).

Patient characteristic	Laparoscopic surgery (n = 139)	Open surgery (n = 175)	*P* value
Age	59.29 ± 12.746	59.11 ± 13.600	.744
Male/Female	81/58	86/89	.107
Weight, kg	58.28 ± 17.619	55.47 ± 13.390	.212
ASA, n (%)			.357
1	17	19	
2	110	130	
3	11	25	
4	1	1	
Previous op history	13 (9.4%)	25 (14.3%)	.183
Procedure			.050
Right hemicolectomy	15	36	
Transverse colon resection	100	107	
Left hemicolectomy	24	32	

ASA = American Society of Anesthesiologists, Op = operation.

**Figure 1.**
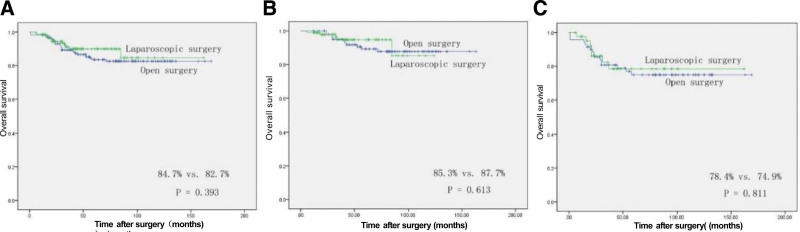
Comparison of 5-year overall survival between the laparoscopic and open groups in all patients (A: 84.7% vs 82.7%, *P* = .393), patients with stage II transverse colon cancer (B: 85.3% vs 87.7%, *P* = .613), and patients with stage III transverse colon cancer (C: 78.4% vs 74.9%, *P* = .811).

**Figure 2.**
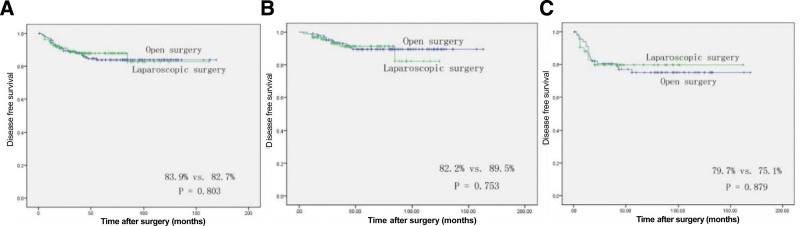
Comparison of 5-year disease-free survival between the laparoscopic and open groups in all patients (A: 82.7% vs 83.9%, *P* = .803), patients with stage II transverse colon cancer (B: 82.2% vs 89.5%, *P* = .753), and patients with stage III transverse colon cancer (C: 79.7% vs 75.1%, *P* = .879).

Table 2 presents the perioperative outcomes of the study. It was observed that laparoscopic approach resulted in a longer operation time compared to open approach (208.96 vs 172.89 minutes, *P* = .044). However, there was no significant difference in intraoperative blood loss between the 2 groups (183.17 vs 192.69 mL, *P* = .987). In the laparoscopic group, a total of 4 cases (2.88%) required conversion to open surgery. Two of these cases were attributed to tight intra-abdominal adhesions, while the other 2 were due to intraoperative bleeding. In the current study, no significant disparity was observed between the laparoscopic and open groups with regards to postoperative recovery, as determined by the duration of time until the occurrence of flatus (4.44 vs 4.06 days, *P* = .470) and the duration of time until the commencement of soft food consumption (4.75 vs 6.20 days, *P* = .074). These findings are consistent with previous studies that have compared the 2 surgical approaches for colorectal cancer. Nonetheless, the laparoscopic method yielded a significantly reduced postoperative hospital stay duration (12.23 vs 14.48 days, *P *= .014) in comparison to the open method. In addition, there was no significant difference in the incidence of postoperative morbidity and mortality between the 2 groups. Regarding pathological outcomes, laparoscopic surgery showed no significant differences in cancer size, number of lymph node retrieval, histologic differentiation, and pTNM stage compared to open surgery (Table 2). The mean follow-up time for the laparoscopic and open surgery groups was 47.22 months and 82.55 months, respectively. Analysis of the 5-year OS rate revealed no statistically significant difference between the laparoscopic (84.7%) and open (82.7%) groups (*P* = .393) (Fig. 1A). Upon performing a stratified analysis based on pTNM stage, no significant discrepancies were observed between the laparoscopic and open groups in patients with stage II (85.3% vs 87.7%, *P* = .613; Fig. 1B) or stage III cancer (78.4% vs 74.9%, *P* = .811; Fig. 1C). Similarly, the 5-year DFS rate was 82.7% in the laparoscopic group and 83.9% in the open group, with no statistical significance (*P* = .803) (Fig. 2A). After conducting a stratified analysis based on pTNM stage, no substantial variances were detected between the laparoscopic and open groups in patients with stage II (82.2% vs 89.5%, *P* = .753; Fig. 2B) or stage III cancer (79.7% vs 75.1%, *P* = .879; Fig. 2C). Overall, our analysis found no significant differences between laparoscopic and open surgery in terms of OS or DFS rates, when stratified by pTNM stage.

**Table 2:** Perioperative outcome (n = 314).

Patient characteristic	Laparoscopic surgery (n = 139)	Open surgery (n = 175)	*P* value
Operation time, minutes	208.96 ± 60.641	172.89 ± 48.173	**.044**
Blood loss, mL	183.17 ± 159.706 (10–1000)*	192.69 ± 271.071 (20–3500)*	.987
Conversion	4 (2.9%)		
Time to flatus, days	4.44 ± 1.435	4.06 ± 1.582	.470
Time to soft food intake, days	4.75 ± 1.923	6.20 ± 3.676	.074
Duration of POD, days	12.23 ± 4.936	14.48 ± 6.597	**.014**
Mortality, n (%)	0	3 (1.7%)	.257
Complications, n (%)	41 (29.5%)	56 (32.0%)	.633
Tumor size, cm	5.41 ± 2.036	5.43 ± 2.377	.163
No of harvested LN	16.10 ± 8.599	13.66 ± 9.692	.886
pT4 stage, n (%)			**.022**
T4a	132	153	
T4b	7	22	
pTNM stage, n (%)			.054
IIB	91	90	
IIC	6	15	
IIIB	30	44	
IIIC	12	26	
Histologic differentiation, n (%)			.356
Well	1	1	
Moderate	118	138	
Poor	20	36	

LN = lymph node, POD = postoperative days; n, number, TNM = tumor-node-metastasis.

* Values are ranges.

Table 3 presents the findings of the univariate analysis, which revealed a significant correlation between OS and various factors. Specifically, differentiation (HR = 3.603, 95% CI: 1.815–7.151), vascular invasion (HR = 0.355, 95% CI: 0.147–0.857), N classification (HR = 5.747, 95% CI: 2.681–12.200), preoperative CA199 (HR = 0.352, 95% CI: 0.176–0.704), and preoperative AFP (HR = 0.120, 95% CI: 0.016–0.886) were significantly associated with OS. Moreover, the multivariate analysis revealed that N classification (HR = 3.435, 95% CI: 1.414–8.264) and differentiation (HR = 2.583, 95% CI: 1.243–5.367) were independent prognostic factors that exhibited a significant association with OS. Similarly, the univariate analysis indicated significant associations between nodal classification (HR = 6.410, 95% CI: 3.086–13.333), vascular invasion (HR = 0.311, 95% CI: 0.137–0.706), differentiation (HR = 2.776, 95% CI: 1.413–5.456), and preoperative CA199 (HR = 0.428, 95% CI: 0.219–0.837) with DFS. However, the multivariate analysis demonstrated that nodal classification (HR = 4.405, 95% CI: 1.984–9.804) was the only independent predictor of DFS. To summarize, the findings of this study suggest that OS is significantly associated with differentiation, vascular invasion, N classification, preoperative CA199, and preoperative AFP. Furthermore, DFS is significantly associated with nodal classification, vascular invasion, differentiation, and preoperative CA199. The multivariate analysis further highlights the independent prognostic significance of N classification and differentiation for OS, and nodal classification for DFS. These results carry significant implications for the clinical management and treatment of individuals affected by this particular disease.

**Table 3:** Prognostic factors for overall and disease-free survival (n = 264).

		OS				DFS			
		Univariate	*P*	Multivariate	*P*	Univariate	*P*	Multivariate	*P*
		HR (95% CI)		HR (95% CI)		HR (95% CI)		HR (95% CI)	
Age (y)			.390				.941		
	≥60								
	<60	0.743 (0.378–1.462)				1.025 (0.541–1.940)			
Gender			.437				.459		
	Male								
	Female	1.311 (0.662–2.597)				1.276 (0.670–2.429)			
T classification			.096				.120		
	T4a								
	T4b	2.030 (0.882–4.672)				1.918 (0.843–4.363)			
N classification			**<.001**		**.019**		**<.001**		**.001**
	N0								
	N1								
	N2	5.747 (2.681–12.200)		3.435 (1.414–8.264)		6.410 (3.086–13.333)		4.405 (1.984–9.804)	
Tumor size (cm)			.533				.687		
	≥6								
	<6	0.807 (0.412–1.583)				0.877 (0.463–1.662)			
Cancer nodules			.964				.770		
	Positive								
	Negative	0.976 (0.344–2.773)				1.167 (0.414–3.290)			
Perineural invasion			.286				.154		
	Positive								
	Negative	0.619 (0.256–1.495)				0.550 (0.242–1.250)			
Vascular invasion			**.021**		.890		**.005**		.283
	Positive								
	Negative	0.355 (0.147–0.857)		0.929 (0.326–2.642)		0.311 (0.137–0.706)		0.610 (0.247–1.506)	
Differentiation			**<.001**		**.011**		**.003**		.053
	Well								
	Moderate								
	Poor	3.603 (1.815–7.151)		2.583 (1.243–5.367)		2.776 (1.413–5.456)		1.936 (0.991–3.785)	
ASA			.413				.578		
	1								
	2								
	3	0.557 (0.228–1.363)				0.910 (0.277–2.985)			
CA199 (ug/L)			**.003**		.108		**.013**		.263
	≥37								
	<37	0.352 (0.176–0.704)		0.534 (0.249–1.146)		0.428 (0.219–0.837)		0.662 (0.321–1.364)	
CA125 (ug/L)			.129				.078		
	≥35								
	<35	0.505 (0.209–1.219)				0.478 (0.211–1.087)			
CEA (ug/L)			.562				.881		
	≥5								
	<5	0.827 (0.435–1.573)				0.956 (0.531–1.722)			
AFP (ug/L)			**.038**		.434		.066		
	≥25								
	<25	0.120 (0.016–0.886)		0.408 (0.043–3.870)		0.155 (0.021–1.134)			
Procedure			.952				.503		
RHC									
TCR									
LHC		1.114 (0.452–2.747)				0.690 (0.251–1.898)			

ASA = American Society of Anesthesiologists, CI = confidence interval, DFS = disease-free survival, HR = hazard ratios, LHC = left hemicolectomy, OS = overall survival, RHC = right hemicolectomy, TCR = transverse colon resection.

## 4. Discussion

Our study is the first and largest to investigate the safety and efficacy of laparoscopic radical surgery for T4 transverse colon cancer.^[20]^ Our current research reveals statistically significant shorter postoperative hospitalization in the laparoscopic approach compared to the open approach, which is consistent with findings from previous studies.^[21–25]^ Although previous studies^[4,26]^ have reported no statistically significant difference in postoperative morbidity between the 2 groups, laparoscopic approach needs superior operative technique for the resection of malignant tumor of the transverse colon. Compared to the open approach, the laparoscopic approach was found to have a statistically longer operative time in the present study, which is consistent with previous studies. The oncological principle governing cancer resection entails the complete removal of malignant tissue with clear surgical margins and subsequent dissection of the systemic lymph nodes. As the tumor size was similar in both groups, and all cases underwent potentially radical resection, our study aimed to compare the 5-year OS and DFS between the laparoscopic approach group and the open approach group. Our analysis revealed that the 5-year overall survival rate was 84.7% in the laparoscopic approach group, while the open approach group demonstrated a slightly lower rate of 82.7%. Similarly, the 5-year DFS was 82.7% and 83.9% in the 2 groups, respectively. Our findings were consistent with previous research and suggest that the oncologic long-term outcomes of the laparoscopic approach are comparable to those of the open approach, as evidenced by the lack of statistically significant differences in 5-year OS (*P* = .393) and DFS (*P* = .803).

Figure S1, http://links.lww.com/MD/K814 shows significant differences in the OS and DFS curves according to the TNM stage. The univariate analysis indicated that differentiation, vascular invasion, nodal classification, preoperative AFP, and preoperative CA199 were significantly associated with OS. Nevertheless, the multivariate analysis demonstrated that both differentiation and nodal classification were independent prognostic factors for OS (both *P* < .05), while other variables did not show significant independent predictive value. Similarly, the results of univariate analysis indicated that differentiation, vascular invasion, N classification, and preoperative CA199 were significantly associated with DFS, whereas only N classification emerged as an independent prognostic factor for DFS based on multivariate analysis (*P* < .05). Moreover, it was observed that T4b transverse colon tumors are more technically challenging than T4a tumors.^[27,28]^ Our findings suggest that the utilization of LC is less common in T4b tumors compared to T4a tumors, potentially due to the involvement of other organs that may require multi-visceral resection. As showed in Figure S2, http://links.lww.com/MD/K815, the 5-year OS was 87.4% for the T4a and 69.3% for T4b stage (*P* = .020), whereas the 5-year DFS were 86.2% and 73.6%, respectively (*P* = .091), which may be explained by latter tumor stage for the T4b tumors.

Compared with the open approach, laparoscopic resection of transverse colon malignancies by experienced surgeons has demonstrated several short-term advantages, including shorter postoperative hospitalization and faster recovery, while maintaining comparable oncologic long-term outcomes. These findings suggest that laparoscopic resection may be a viable alternative for the treatment of transverse colon malignant tumors. However, it is important to consider the limitations of this retrospective study, including potential sample selection bias. While the sample size in this investigation was larger than previous studies, the infrequency of transverse colon malignancy presents a challenge in conducting randomized controlled trials, which are fundamental in establishing conclusive results. Despite the limitations imposed by the constraints of our study, our findings offer valuable evidence endorsing the application of laparoscopic radical surgery in treating malignant tumors located at pT4 stage in the transverse colon. Further randomized controlled trials are needed to assess the efficacy and safety of laparoscopic resection for pT4 TCC.

## 5. Conclusion

The research has indicated that laparoscopic radical resection for TCC offers a number of perioperative advantages and does not reduce the long-term prognosis of the tumor relative to open approach. Laparoscopic approach for T4 TCC was correlated with shorter postoperative hospitalization. Therefore, considering the evidence presented, laparoscopic approach could potentially serve as a viable alternative for radical resection of pT4 transverse colon cancer.

## Author contributions

**Conceptualization:** Feng Xie, fan Ping Lu, ming Yu Chen, jun Xiang Liu, Zhenhong Zou, heng Jin Gan.

**Data curation:** Feng Xie, fan Ping Lu, ming Yu Chen, jun Xiang Liu, Zhenhong Zou.

**Investigation:** Feng Xie, fan Ping Lu, ming Yu Chen, jun Xiang Liu, heng Jin Gan.

**Methodology:** Feng Xie, fan Ping Lu, ming Yu Chen, jun Xiang Liu, heng Jin Gan.

**Project administration:** Feng Xie, ming Yu Chen, jun Xiang Liu, Zhenhong Zou.

**Resources:** Feng Xie, fan Ping Lu.

**Software:** Feng Xie, fan Ping Lu.

**Supervision:** Zhenhong Zou.

**Writing – original draft:** Feng Xie.

**Writing – review & editing:** Feng Xie, Zhenhong Zou, heng Jin Gan.

## Supplementary Material




