# Supplementary material for: Laparoscopic radical surgery for locally advanced T4 transverse colon cancer and prognostic factors analysis: Evidence from multi-center databases
Source: Medicine (Baltimore). 2023 Dec 1;102(48):e36242. doi: 10.1097/MD.0000000000036242 (PMC10695505; doi:10.1097/MD.0000000000036242)
Supplement: Supplementary file 1 [file medi-102-e36242-s001.doc]

Figure S2 OS and DFS curves according to the TNM stage. OS=overall survival,DFS=disease-free survival.


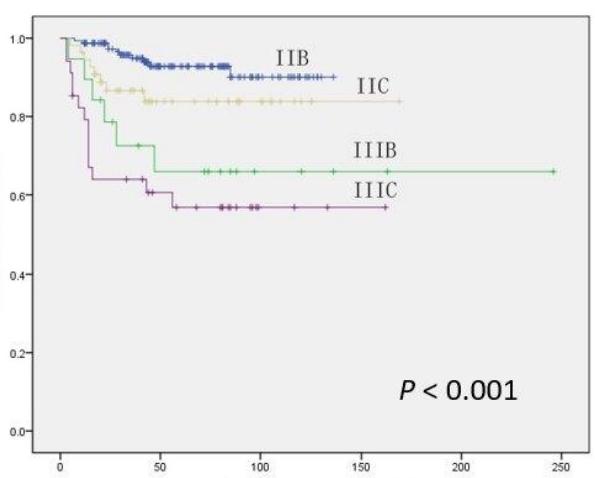


A

Ove ra l l survial


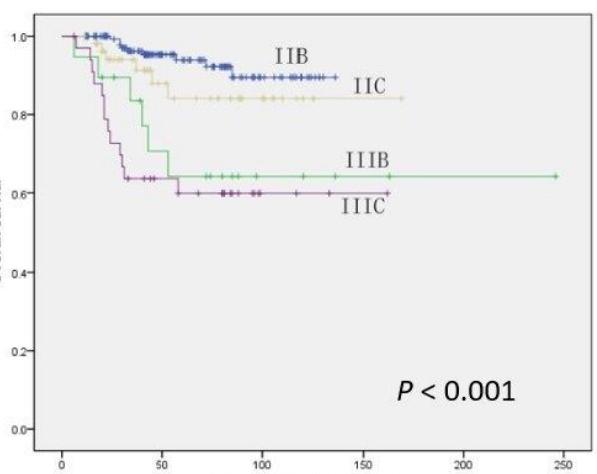


**Time** **after** **surgery** **(months)**

B

Di sease f ree survival

**Time** **after** **surgery** **(months)**
