# Supplementary material for: Laparoscopic radical surgery for locally advanced T4 transverse colon cancer and prognostic factors analysis: Evidence from multi-center databases
Source: Medicine (Baltimore). 2023 Dec 1;102(48):e36242. doi: 10.1097/MD.0000000000036242 (PMC10695505; doi:10.1097/MD.0000000000036242)
Supplement: Supplementary file 2 [file medi-102-e36242-s002.doc]

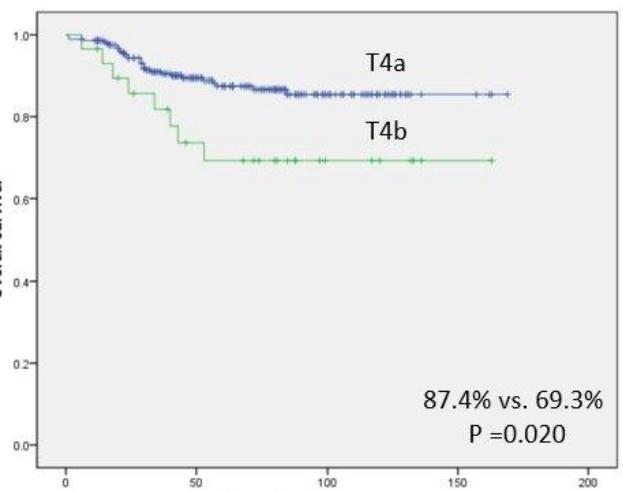


Figure S2 Comparison of 5-year OS and DFS between the T4a and T4b groups for transverse colon cancer. OS=overall survival,DFS=disease-free survival.


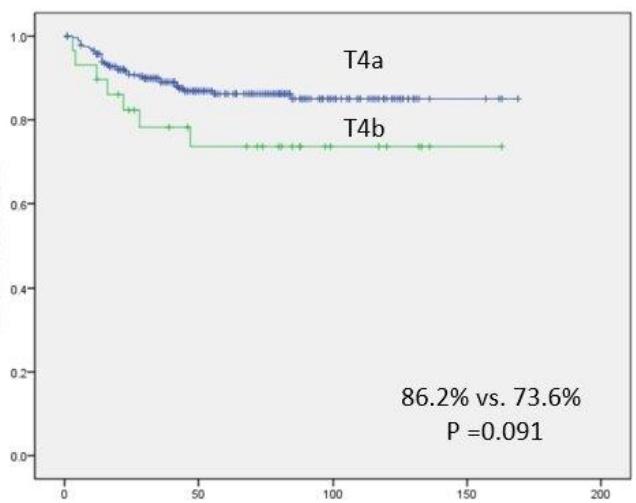
A

B

Ove ra l survival

Disease free survival

**Time** **after** **surgery** **(months)**

**Time** **after** **surgery** **(months)**
